# The Sphygmographer, or Register of the Arterial Pulse

**Published:** 1860-07

**Authors:** 


*The Sphygmographer, or Register of the Arterial Pulse.*—M. Marey has published in the *Gazette Medicale*, an article wherein he describes an improvement on Vierodt's arterial register. The French instrument also acts by a lever, but is lighter and of easier practical application. It can only indicate the frequency or the more or less regularity of the pulse. It may be doubted whether these instruments, though very ingenious, will ever prove actually useful in practice.

